# Cryptic population structure at the northern range margin of the service tree *Sorbus domestica*

**DOI:** 10.7717/peerj.14397

**Published:** 2022-12-05

**Authors:** Georg F.J. Armbruster, Kay Lucek, Yvonne Willi

**Affiliations:** Department of Environmental Sciences, University of Basel, Basel, BS, Switzerland

**Keywords:** Climate change, Drought-adapted, Genetic diversity, Forestry, Rare trees, Population genetics

## Abstract

Climate change has aroused interest in planting warm- and drought-adapted trees in managed forests and urban areas. An option is to focus on tree species that occur naturally, but have centers of distribution in warmer and drier areas. However, in order to protect the species pool of genetic diversity, efforts of planting and promotion should be informed by knowledge on the local genetic diversity. Here, we studied the macro- and micro-scale population genetic structure of the rare European fruit tree *Sorbus domestica* at its northern range margin, in western Switzerland. New microsatellite data were combined with published data from across the European distribution of the species. Analyses revealed the presence of mainly one of two species-wide ancestral clusters, *i.e.*, the western European cluster, with evidence that it consists of two cryptic sub-clusters. Average pairwise *F*_ST_ of 0.118 was low across the range, and only allelic richness was reduced in the northern margin compared to more southern and southeastern areas of Europe. Based on our finding of considerable genetic diversity of the species in western and northern Switzerland, we suggest that a national propagation program should focus on collecting seeds from natural, high-density tree stands and propagate locally. More generally, our study shows that rare tree species in marginal areas of their distributions do not necessarily have low genetic diversity or heightened levels of inbreeding, and in those cases probably need no assisted migration in efforts to propagate them.

## Introduction

Organisms with long generation times such as tree species are especially vulnerable under rapid climate warming (Smith & Beaulieu, 2009; Dauphin et al., 2021). In forestry practice, this has been recognised and started being countered by efforts of assisted migration, the planting of native trees from *e.g.*, hotter or drier provenances or the planting of non-native species from hotter and drier climates (Williams & Kasten Dumroese, 2013). Another option is the planting and promotion of generally hot- and dry-adapted species native to an area that is otherwise (so far) their cool or wet margin of species distribution. This approach of tree management benefits from detailed knowledge on the spatial scale of intraspecific genetic connectivity and population structure as two important aspects of conservation genetics (Sork & Smouse, 2006; Hmeljevski et al., 2017). Successful conservation management requires an understanding of the biogeographic context of the populations in question *e.g.*, to avoid the potential introduction of maladaptive alleles or the replacement of a locally adapted lineage with an introduced one (Hoban et al., 2016). The latter is especially true for tree species that have also been cultivated, in which human-mediated gene flow and species propagation have shaped both the current population structure and the distribution (Krutovsky et al., 2012; Khoury et al., 2022).

An example of such species are various fruit trees in Europe, that have their centres of species distribution in generally hot and dry climates. There is growing interest in propagating these species in central Europe, which so far has been the cold and wet margin of their distributions (Goldschmidt, 2013). The biogeographic and spatial scale of population genetic structure of insect-pollinated fruit trees in Europe often varies over a wide range, from regional over national to continental (Table 1). Different factors may contribute to the observed variation among species. One factor is the cultivation history of the investigated species and samples, ranging from wild tree stands, *e.g.*, in the wild service tree *Sorbus torminalis* (Kučerová et al., 2010), to semi-natural and cultivated stands, *e.g.*, in the sweet cherry *Prunus avium* (Barreneche et al., 2021). The population genetic structure may be further affected by clonality, as suggested for wild cherry stands (Stoeckel et al., 2006), or by inbreeding (Kamm et al., 2009; Cornille et al., 2012; Gross et al., 2014; Table 1). The level of genetic differentiation may also differ, which has been attributed to isolation-by-distance, restricted gene flow by pollinators, landscape and habitat structure, past human-mediated cultivation and/or genetic drift (Angelone et al., 2007; Kramer et al., 2008; Yuan, Cheng & Zhou, 2011; Cornille et al., 2015; VanStrien, Holderegger & Van Heck, 2015). Many studies also aimed to infer the number of genetic clusters within a given dataset. At a broad geographic scale, such clusters often define distinct postglacial-recolonization lineages, especially when wild tree stands were investigated (Cornille et al., 2013; GenTree, 2020). However, additional fine-scale population structure is often observed at a smaller geographic scale that can be used to inform national management strategies (Muccillo et al., 2019).

**Table 1:** Microsatellite studies on various insect-pollinated, European fruit tree taxa of wild and natural stands (W), semi-natural stands (S) and samples from cultivars (C) revealed ranges of inbreeding coefficients (*F*_IS_), of differentiation coefficients (*F*_ST_) among stands and the best supported number of genetic clusters (K) obtained by STRUCTURE analysis.

**Taxon**	**Region**	**Samples**	*F*_**IS**_	*F*_**ST**_	**Best K**
Chestnut, *Castanea sativa* Fagaceae [1]	Switzerland	S, C	0.12	0.03–0.10	2
Chestnut *Castanea sativa* Fagaceae [2]	Spain	W, S, C	-0.23–0.29	∼0.14	3
Pear *Pyrus sp.* Rosaceae [3]	Europe, Middle East, Eastern Asia	C	0.06–0.15	0.01	3–6
Sweet cherry *Prunus avium* Rosaceae [4]	Italy (region of Campania)	C	-0.76	0.50	3
Sweet cherry *Prunus avium* Rosaceae [5]	Europe	S, C	-0.02 – -0.01	–	4
Sweet cherry *Prunus avium* Rosaceae [6]	Europe, Turkey, Iran	W, S, C	-0.09–0.06	0.01–0.11	2–4
Apple *Malus sylvestris* Rosaceae [7]	Europe	W	0.07–0.10	0.04–0.12	2–6
Apple *Malus sylvestris* Rosaceae [8]	Slovenia	W, C, hybrids	-0.28–0.14	0.08–0.38	2
Checker tree *Sorbus torminalis* Rosaceae [9]	Eastern Europe, with the Balkans, Caucasus	W	–	0.23	5
Service tree *Sorbus domestica* Rosaceae [10,11]	Europe, excluding Switzerland	W, S, C	-0.05	0.14	3

**Notes.**

References: [1], Pereira-Lorenzo et al. (2020); [2], Martín et al. (2012); [3] Ferradini et al. (2017); [4], Muccillo et al. (2019); [5], Barreneche et al. (2021); [6], Mariette et al. (2010); [7], Cornille et al. (2013); [8], Kišek, Jarni & Brus (2021); [9], Kučerová et al. (2010); [10], George et al. (2015); [11], George et al. (2016).

Here we focused on the population structure of the service tree *Sorbus domestica* L. (Rosaceae) in western and northern Switzerland. The service tree is a Mediterranean species occurring from Spain to northwestern Turkey, reaching its northern distribution limit in central Europe (Kausch-Blecken von Schmeling, 2000; Špíšek, Otto & Vašut, 2021). It tends to grow in open, sun-exposed deciduous forests. The species has been cultivated since the Roman times both for its fruits and timber (Rotach, 2003), and this history has influenced its northern distribution by human-mediated dispersal through cultivation and forest management over the last two millennia (Kausch-Blecken von Schmeling, 2000; Hrdoušek et al., 2014). In central Europe, it is generally a rare species and therefore listed as locally endangered, *e.g.*, in Switzerland (InfoFlora, 2022). While the reason may be the so far mostly marginal climate, changes in forest management and population fragmentation may have contributed to its rarity (Rotach, 2003). In Switzerland, the species was reported to hardly propagate by seeds, which has been attributed to changing forest management affecting seed germination rate (Kamm et al., 2011; Kamm et al., 2012). And, while the service tree is an insect-pollinated species that produces outcrossed seeds, a selfing rate of more than 30% was observed in low-density stands, together with clonal reproduction by root suckers (Rotach, 2003; Kamm et al., 2012).

Our main research question was whether the species was genetically isolated and impoverished in western and northern Switzerland relative to more central parts of species distribution. A former biogeographic study had suggested three genetic clusters for *S. domestica* across Europe, *i.e.,* a Mediterranean/Balkan, a western (France) and an eastern (Austria) cluster (see Table 1, George et al., 2015). However, the aforementioned study lacked samples from Switzerland, precluding an assessment of the biogeographic context of the extant distribution of this species. Combining previously published genetic data with newly genotyped individuals from Switzerland, we reconstructed the population structure of service trees across Switzerland and related it to the broader biogeographic context of this species. We finally bridged between a micro-scale, Swiss study of Kamm et al. (2009) and the macro-scale, European assessment of population structure in *Sorbus domestica* (George et al., 2015; George et al., 2016) to inform about conservation efforts on a national scale.

## Material & Methods

Our dataset comprised both newly genotyped individuals from southwestern and northwestern Switzerland and published data from three studies covering northeastern Switzerland (Kamm et al., 2009) and Europe (George et al., 2015; George et al., 2016). For the newly genotyped individuals, fresh leaf samples of 82 GPS-referenced, mature trees from southwestern (*N* = 9) and northwestern Switzerland (*N* = 73) were collected in 2020 (Table S1). Some of the leaf samples were collected in a nursery in Biel, Switzerland, from trees for which the original topographic sampling localities of the grafted tree-material were known. Leaf samples were frozen and stored at -20 °C. We then disrupted parts of the frozen leaf samples (∼1.5 cm^2^) in liquid nitrogen, followed by immediate DNA extraction using the DNeasy Plant Mini Kit (Qiagen, Hilden, Germany). We genotyped all individuals at the seven microsatellite loci used by Kamm et al. (2009), George et al. (2015) and George et al. (2016), *i.e.,* MSS5, MSS16, CH01h10, CH01h01, CH02c09, BGT23b and MS14H03 (Fig. S1A). The multiplexing protocol and PCR cycling conditions were taken from Kamm et al. (2009). Microsatellite fingerprinting and allele scoring were performed by the company ECOGENICS (Balgach, Switzerland). Fingerprinting and scoring were repeated for a total of 10 individuals, providing an overlapping allele assignment of 97.5% over all loci, *i.e.,* a low error rate of 2.5%. No allelic dropouts occurred, indicating low or no null-allele abundance.

We re-genotyped leaf samples of three individuals included in Kamm et al. (2009) and of 12 individuals of southeastern Bulgaria of George et al. (2015). This allowed us to standardize the locus-specific amplicons for each dataset (Fig. S1B). We then omitted individuals with missing data for more than one locus. This resulted in microsatellite data for 162 trees of northeastern Switzerland and 356 trees from 16 sites across Europe, without Switzerland, which we merged with the newly-collected genotype data from western Switzerland.

In a preliminary analysis on the data, we identified 33 local clones with identical multi-locus genotypes (Table S2) using GenAlEx 6.51b2 (Peakall & Smouse, 2012). For these, we only included one randomly selected tree of each clone in our final dataset. The final new data therefore comprised the following sample size: nine trees from southwestern Switzerland, 63 trees from northwestern Switzerland, 152 trees from northeastern Switzerland and 310 trees of 16 populations from other regions of Europe, summing up to an overall sample size of 534 trees (Table S3). We started analyses with assessing patterns of population structure with STRUCTURE 2.3.1 (Pritchard, Stephens & Donnelly, 2000) on all individuals from across Europe. Because potential substructure can be masked in large and/or unbalanced datasets (Wang, 2017), we also ran STRUCTURE for Swiss samples combined with individuals from geographically close sites from Austria, France and Italy, and, finally, Swiss samples only. In each case, we ran STRUCTURE under the admixture model with 200’000 MCMC steps as burn-in, followed by 1’000’000 iterations. We assumed 1 to 15 genetic clusters with ten repeats for each K. We identified the statistically best fitting K for each dataset using Structure Harvester (Earl & VonHoldt, 2012), which implements the method of Evanno, Regnaut & Goudet (2005). We further conducted a principal component analysis (PCA) with GenoDive 3.0 (Meirmans, 2020) on all 534 tree individuals.

To compare the level of between- and within-population genetic variation of *Sorbus domestica* from western and northern Switzerland with samples from across the species range, we first calculated population differentiation (*F*_ST_) among all European localities with 10’000 permutations. We estimated allelic richness (*AR*) by rarefaction for each locality with at least 10 individuals with FSTAT 2.9.4 (Goudet, 1995). For each sampling location, we estimated the observed (*H*_o_) and expected heterozygosity (*H*_e_), the number of alleles, the inbreeding coefficient (*F*_IS_) and the number of private alleles by using GenAlEx. Finally, we tested for isolation-by-distance by performing a Mantel test between pairwise geographic and Euclidean genetic distances among individuals within Switzerland. Testing was performed using the package *vegan* (Dixon, 2003) in R 4.1.1 (R Core Team, 2021) with 1’000 bootstrap replicates to assess significance.

## Results

**Figure 1:**
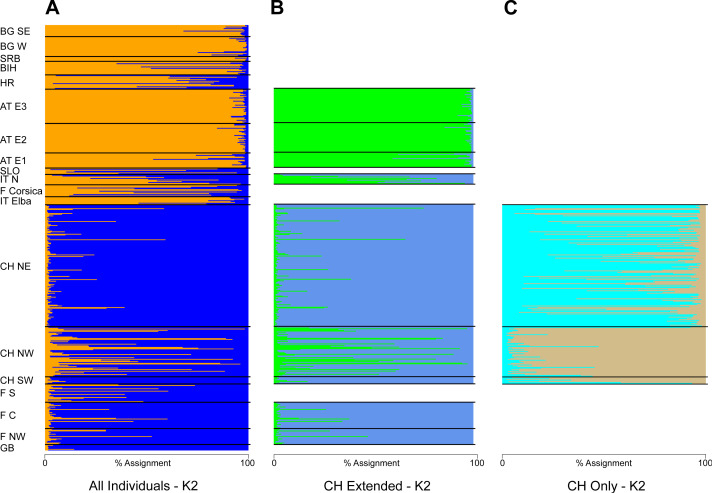
Summary of the individual-based cluster-assignment analysis using STRUCTURE. Including either (A) all individuals from across Europe (localities abbreviated as in Table 2), (B) individuals from Switzerland and neighboring countries or (C) Switzerland only. Shown is the assignment (in percent) for K = 2, the best supported number of genetic clusters for all datasets.

**Figure 2:**
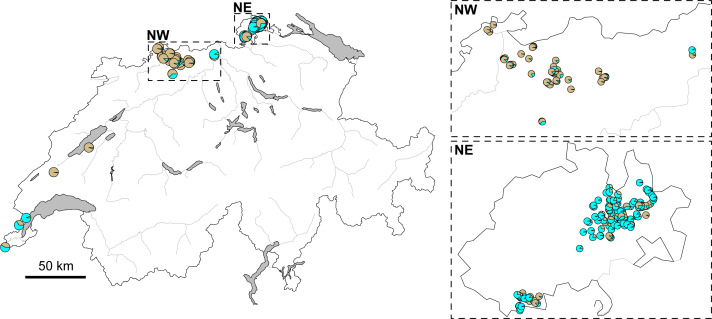
Map of Switzerland with pie charts indicating cluster assignment based on STRUCTURE results. For all genotyped individuals of Switzerland, from near Lake Geneva on the lower left to the area west of Lake Constance on the upper right (Fig. 1C). Inlets focus on the northwestern (NW) and northeastern (NE) regions, respectively. Major rivers are indicated in gray. (Map data adopted from the Federal Statistical Office, Neuchâtel, Switzerland).

The STRUCTURE analysis identified two genetic clusters (K = 2) as the best supported scenario for the complete dataset (Fig. S2), separating populations broadly in a western and an eastern genetic cluster (Fig. 1A). Here, individuals from the southwestern, northwestern and northeastern parts of Switzerland were all assigned to the western cluster. Similar assignments were obtained when individuals from Switzerland and geographically close populations were included, with the best K being also 2 (Fig. 1B, Fig. S2). Cryptic substructure within Switzerland became though apparent when only Swiss individuals were included (Fig. 1C, Fig. S2), supporting two genetic clusters associated with the northwestern and northeastern part of the distribution, respectively, with signatures of gene flow between them. Interestingly, individuals from southwestern Switzerland were assigned to both clusters in the analysis of this national dataset (Figs. 1C and 2).

**Figure 3:**
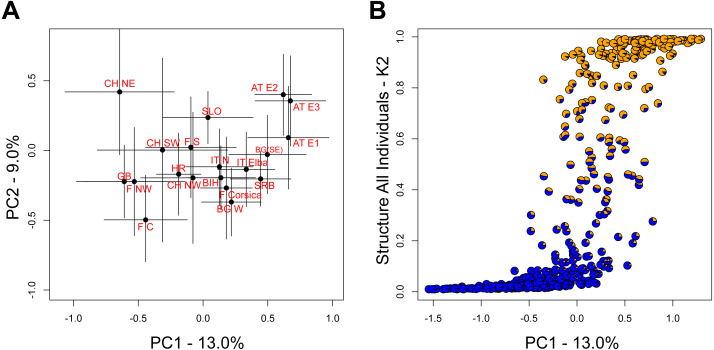
Relationship among populations based on principal components (PC). (A) Average PC scores for each population (±1 SD) along the first and second PC axes. (B) Relationship between the scores of the first PC axis for each individual and the overall STRUCTURE assignment (K = 2, Fig. 1A).

The two leading principal component (PC) axes for the complete dataset accounted for 13.0% and 9.0% of the total variation, respectively. While populations generally sorted out by longitude along PC1, there was substantial variation within populations (Fig. 3A). Nevertheless, the differentiation along PC1 was consistent with the aforementioned STRUCTURE analysis on all individuals (Fig. 3B).

**Figure 4:**
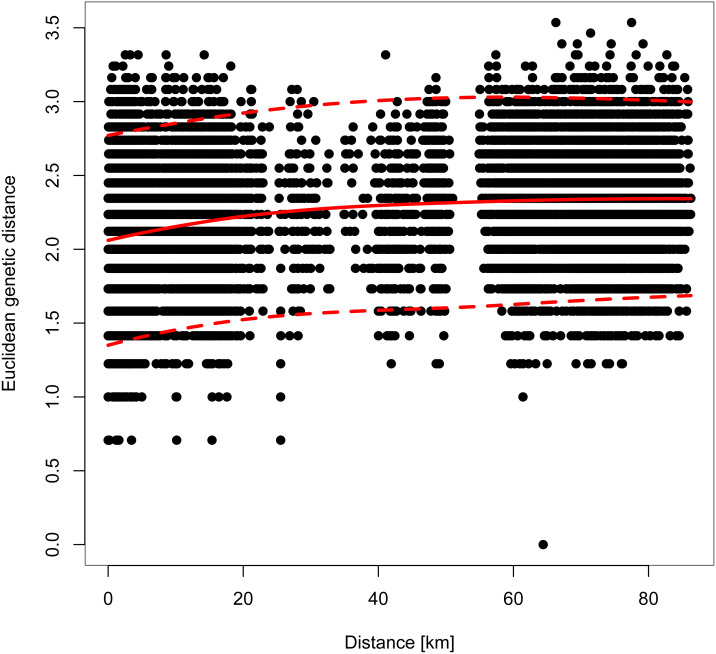
Pairwise relationship between the individual-based Euclidean genetic distance and the geographic distance. For all Swiss individuals except samples from southwestern Switzerland. A Mantel test suggested a significant contribution of isolation-by-distance. The solid red line indicates the model fit of a local polynomial regression, and the dashed lines outline the 95% confidence limits.

However, genetic differentiation was generally low across the 19 European localities, with the average pairwise genetic differentiation (*F*_ST_) being 0.118 (range -0.004–0.253, Table S4). Within Switzerland, performing an isolation-by-distance test suggested a significant relationship between genetic and spatial distances across individuals from northwestern and northeastern Switzerland (*r*_MANTEL_ = 0.289, *p* = 0.001; Fig. 4). This relationship became weaker when individuals from southwestern Switzerland were also included (*r*_MANTEL_ = 0.161, *p* = 0.001), consistent with the presence of both genetic clusters in this region (see Fig. 2).

**Figure 5:**
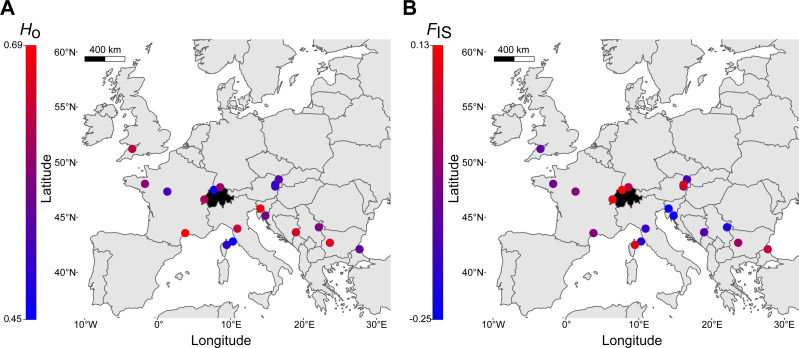
Distribution of genetic diversity of *Sorbus domestica* across Europe. (A) Observed heterozygosity, *H*_*o*_, (B) inbreeding coefficient, *F*_IS_ (see Table 2 for details). Switzerland is highlighted in black.

Finally, a comparison of within-locality genetic diversity revealed that the three Swiss sites had allele numbers, allelic richness, observed heterozygosity (Fig. 5A) and expected heterozygosity well within the ranges of other European regions (Table 2). *F*_IS_ had an overall mean of -0.03 (range: -0.25–0.13, Fig. 5B), with the highest *F*_IS_ occurring in northwestern Switzerland, suggesting a deviation from Hardy-Weinberg equilibrium (Table 2). Allelic richness was the only genetic parameter that was significantly different between localities of the northern margin (*N* = 9) and localities of the southern and southeastern species range (*N* = 10), with northern localities having lower allelic richness (*t*-test, *p* = 0.016; *H*_o_, *H*_e_, *F*_IS_ with *p* > 0.05). The number of alleles identified across the seven loci was 88 (Table 2). Exclusive private alleles occurred generally at low frequencies (<0.06), with the exception of Serbia and Bulgaria, ranging between 0.125 and 0.200 (Table 2).

## Discussion

The population genetic structure of forestry and cultivated tree species is shaped by past and current human-mediated dispersal and management. While its assessment is crucial to inform conservation and forestry management, the geographic scale at which it is quantified is often too broad or too narrow for a national conservation strategy (Table 1). This was the case for the service tree *Sorbus domestica* in Switzerland. Our study bridges between former research of this species that was either done at a continental (George et al., 2015; George et al., 2016) or at a local scale (Kamm et al., 2009). By compiling the spatially broadest dataset for this species, we show that (i) the individuals fall into two genetic clusters at a continental scale with substantial admixture between the two (Figs. 1A and 3B) and that (ii) there is cryptic population differentiation north of the Alps, *i.e.,* between northwestern and northeastern Switzerland, and again with substantial admixture (Figs. 1C, 2 and 4). We discuss these findings in the context of possible past demographic processes and both broad-scale and local-scale conservation implications, with insights likely applicable to many similarly managed tree species (*e.g.*, *Sorbus torminalis*, see Angelone et al., 2007; Jankowska-Wroblewska, Warmbier & Burczyk, 2016).

### Continental European structure

The biogeographic history of many European species is shaped by postglacial expansion from one or multiple glacial refugia (Taberlet et al., 1998; Hewitt, 1999; Schmitt, 2007; Conord, Gurevitch & Fady, 2012). Indeed, genetic clustering along a longitudinal gradient from west to east has been found in several natural and managed taxa (Schmitt, 2007; Conord, Gurevitch & Fady, 2012; Cornille et al., 2015). While in *S. domestica* human-mediated dispersal and long-term cultivation has been suggested to also have played a role in the current distribution patterns (Kausch-Blecken von Schmeling, 2000; Hrdoušek et al., 2014), our findings are consistent with two major biogeographic lineages across Europe in this species (Fig. 1A). This somewhat contrasts with the former continent-scale assessment (George et al., 2015), which suggested three major genetic clusters (Table 1). Two factors may account for this difference: First, our compiled dataset comprises more sampled sites, which bridge the former collection gap in central Europe (Table 2). Second, we note that George et al. (2015) did not fully explore the parameter space of their population structure analysis by restricting their inferences to only two to four genetic clusters. However, to assess the likelihood of two genetic clusters, the method by Evanno, Regnaut & Goudet (2005) requires the statistical inference assuming only one genetic cluster (Porras-Hurtado et al., 2013; Wang, 2017). The overall population structure that we observed was similarly reflected in results of the principal component analysis, with populations sorting out by longitude on the first axis (Fig. 3A). However, this does not exclude further, often more subtle regional substructure or the possibility for translocations, the latter being indicated *e.g.*, in parts of France (Fig. 1A) or the Czech Republic (Špíšek, Otto & Vašut, 2021). While the latter study found remarkable substructure, it is likely that the investigated trees belonged to the eastern cluster.

**Table 2:** Microsatellite diversity in *Sorbus domestica* of marginal/northern areas and of southern areas of the species distribution.

**ID**	**Sampling locality**	**Source**	***N***	**# Alleles**	***AR***	***H***_**o**_	***H***_**e**_	*F*_**IS**_	**SE** *F*_**IS**_	**Private alleles**
CH SW	Southwestern Switzerland	This study	9	4.6	–	0.63	0.65	0.06	0.121	0
CH NW	Northwestern Switzerland	This study	63	7.6	4.0	0.54	0.62	0.13	0.029	2 (0.008, 0.008)
CH NE	Northeastern Switzerland	[1]	152	6.1	3.6	0.62	0.64	0.02	0.027	3 (0.003, 0.003, 0.010)
GB	Southwestern Great Britain	[2]	7	4.0	–	0.65	0.63	-0.04	0.098	0
F NW	Northwestern France	[3]	20	4.7	3.8	0.62	0.6	-0.04	0.051	0
AT E1	Eastern Austria	[3]	19	4.9	3.8	0.58	0.54	0.00	0.112	0
AT E2	Eastern Austria (Wolkersdorf)	[3]	37	5.6	3.8	0.6	0.58	-0.05	0.072	0
AT E3	Eastern Austria (Merkenstein)	[3]	43	5.4	3.6	0.54	0.56	0.03	0.022	1 (0.047)
SLO	Slovenia	[3]	8	3.4	–	0.68	0.53	-0.25	0.047	0
*Mean*	*Northern range margin*		–	*5.1*	*3.8*	*0.61*	*0.59*	*-0.02*	* 0.064*	*–*
HR	Croatia	[3]	18	4.6	3.8	0.61	0.53	-0.15	0.033	2 (0.056, 0.029)
F C	Central France	[3]	33	5.3	3.9	0.59	0.59	-0.02	0.037	0
F S	Southern France	[3]	23	5.3	4.1	0.69	0.68	-0.03	0.036	0
IT Elba	Island of Elba, Italy	[3]	10	3.4	3.8	0.45	0.46	-0.06	0.090	0
F Corsica	Island of Corsica, France	[3]	15	5.1	4.2	0.57	0.61	0.04	0.041	0
IT N	Northern Italy	[3]	13	5.3	4.4	0.65	0.61	-0.05	0.079	1 (0.038)
BIH	Bosnia and Hercegovina	[3]	17	5.7	4.5	0.65	0.62	-0.05	0.072	0
SRB	Serbia	[3]	6	4.3	–	0.61	0.56	-0.09	0.058	2 (0.200, 0.100)
BG W	Western Bulgaria	*	25	7.3	5.0	0.66	0.65	-0.02	0.042	3 (0.152, 0.020, 0.083)
BG SE	Southeastern Bulgaria	[3]	16	6.0	4.7	0.60	0.62	0.02	0.061	2 (0.063, 0.125)
*Mean*	*Southern and southeastern range*	–		*5.2*	*4.3*	*0.61*	*0.59*	*-0.04*	*0.055*	–
Overall mean		–		*5.2*	*4.1*	*0.61*	*0.59*	*-0.03*	*0.059*	–

**Notes.**

The table lists: code for locality, ID; number of trees, N; average number of alleles across seven loci; average allelic richness rarefied to seven individuals, *AR*; average observed heterozygosity, *H*_o_; average expected heterozygosity, *H*_e_; average inbreeding coefficient with standard error (*F*_IS_, SE); and the number of private alleles (with respective frequencies). Only samples from CH SW and CH NW were newly genotyped for this study; sources of genetic data for other localities are listed. [1], Kamm et al. (2009); [2], George et al. (2016); [3], George et al. (2015); * Unpublished data provided by Jan-Peter George.

The two European *Sorbus domestica* lineages are not reproductively isolated as indicated by evidence of substantial admixture between them (Figs. 1A and 3B). This is consistent with several other tree species (GenTree, 2020), for which hybrids between different lineages can be commonly found in their respective contact zones. Regular gene exchange among localities was also suggested by the low level of average pairwise genetic differentiation across Europe (*i.e., F*_ST_ < 0.10; Table S4) and good outcrossing within stands by the generally negligible levels of inbreeding (*F*_IS_; Table 2). Furthermore, we found little evidence for a reduction in within-locality genetic diversity in the northern range margin compared to southern and southeastern areas of distribution. An exception was allelic richness, which decreased from southern areas towards northern areas, as is often found for postglacial range expansions (Table 2; Hewitt, 1999; Schmitt, 2007). Together, these observations could reflect long-distance pollen flow and seed dispersal in *S. domestica* (Kamm et al., 2009; Kamm et al., 2011; Špíšek, Otto & Vašut, 2021), likely combined with long-term human management preventing the emergence of considerable population structure.

### Cryptic population structure in Switzerland

In Switzerland, stands of both *S. domestica* and the sibling species *S. torminalis* are declining in woodlands as a result of too dense canopies and a lack of sun-exposed patches, which are necessary for seed germination (Kamm et al., 2009; Kamm et al., 2011). Because *S. domestica* is considered to be locally endangered in Switzerland, a national seed propagation program was established, that uses seeds from southwestern and northeastern regions (WSL, 2022), but not from the genetically distinct northwestern part of the country. However, former genetic analyses were only done at a local scale, *i.e.,* within northeastern Switzerland (Kamm et al., 2009), precluding the identification of potential genetic structure at a broader scale. Our analyses suggest that such genetic structure indeed occurs and falls along a longitudinal gradient (Fig. 1C) with significant isolation-by-distance (Fig. 4). The two clusters that we identified showed though only subtle genetic differentiation (*F*_ST_ = 0.046, *p* < 0.0001; Table S3), indicating some gene flow between them. Gene flow in *Sorbus domestica* may be attributed to historic human-mediated seed transfer whilst current gene flow among populations is likely limited, given that there are only few and isolated trees connecting the northwestern and northeastern clusters (InfoFlora, 2022). A lack of connectivity has also been suggested to cause cryptic population structure in Austrian *S. domestica* (George et al., 2015) and in *S. torminalis* in Switzerland (Angelone et al., 2007).

However, also some inbreeding may have contributed to cryptic population structure. The tree stands in northwestern Switzerland showed the highest level of inbreeding (Table 2). Leaf samples for this area were taken from wild tree stands of natural deciduous forest, but also from old and cultivated trees of mixed orchards and urban sites. Increased *F*_IS_ among trees may have been produced by habitat fragmentation and by the local propagation of trees. Furthermore, individual-based assignment analysis suggested that individuals from the second major European lineage could have been introduced in this area of Switzerland and gone through separate founder events, resulting in a Wahlund effect, *i.e.,* a subpopulation structure increasing overall *F*_IS_.

Despite the marginal position of Switzerland in the distribution of *S. domestica*, we found considerable within-population genetic diversity (Table 2), including in northwestern Switzerland. This mirrors a potential genetic reservoir for future seed propagation in forestry management.

## Conclusions

Taken together, our findings have implications for the national conservation strategy of *S. domestica*, particularly in light of promoting the species because of its heat and drought tolerance. First, because the tree stands of northwestern Switzerland belong to a cryptic cluster, we recommend that seeds of the northwestern region should also be included to preserve genetic diversity at a national scale. Second, seed propagation should be administered locally or regionally to prevent admixture with foreign gene pools. Third, because low density of trees favors high self-fertilization, *e.g.*, in isolated trees, seeds should be collected from individual-rich and dense natural tree stands (Kamm et al., 2012). This is because self-fertilized seeds are less vital and show a trend towards albinism (*i.e.,* the cotyledons have a lack of chlorophyll, see Kamm et al., 2012). Fourth, trees should be checked for clonality, particularly if nearby trees are of similar size and age. These recommendations could further help increase the standing genetic diversity of the northwestern stands. More generally, our study highlights that cryptic genetic substructure may be predominant even in managed species, requiring more fine-scale management strategies at a national level.

##  Supplemental Information

10.7717/peerj.14397/supp-1 Table S1 Summary of the 82 newly genotyped Sorbus domestica trees from Switzerland, with local clones in different colours

10.7717/peerj.14397/supp-2 Table S2 Number of clones (identical multi-locus genotypes) found at the sampling localities (see Table 2)

10.7717/peerj.14397/supp-3 Table S3 Summary of the 534 *Sorbus domestica* trees from Europe including the new samples from Switzerland Genotype data for the 7 microsatellite markers adjusted to the binning of the new Swiss samples. Sources of genetic data are listed. [1], Kamm et al. (2009); [2], George et al. (2016); [3], George et al. (2015); * Unpublished data provided by Jan-Peter George.

10.7717/peerj.14397/supp-4 Table S4 Pairwise *F*st values (lower left of diagonal) between sampling localities (populations) and their probability, *p* based on 9999 permutations (upper right of diagonal) Significant *p*-values after Bonferroni-correction of alpha (0.05/171 = 0.0003) are highlighted in bold. The full names of localities are presented in Table 2.

10.7717/peerj.14397/supp-5 Figure S1 Allele frequency distribution and their respective adjustments A—Allele frequencies for all newly genotyped individuals from CH NW. B—Allele frequencies of the three re-genotyped individuals used by Kamm et al. (2009) from CH NE. Green—original genotypes, black—genotypes of re-genotyped individuals. C & D—Allele frequencies for F C and IT N, respectively. Green—original genotypes, gray—adjusted genotypes based on 12 re-genotyped individuals used by George et al. (2015). Numbers indicate the shift in base pairs (bp) for each marker. See Table 2 in the main text for details about the populations.

10.7717/peerj.14397/supp-6 Figure S2 Delta K values on number of clusters K for the three datasets analyzed A—Optimum at K=2 for the dataset including all European samples (Fig. 1A). B—Optimum at K=2 for the dataset including samples from Switzerland and surrounding areas (Fig 1B). C—Optimum at K=2 for the dataset including Swiss samples only (Fig. 1C).

## Additional Information and Declarations

### Data Availability

The following information was supplied regarding data availability: The genotype (microsatellite) data is available in the Supplemental Files.
