# Supplementary material for: Cryptic population structure at the northern range margin of the service tree Sorbus domestica
Source: PeerJ. 2022 Dec 5;10:e14397. doi: 10.7717/peerj.14397 (PMC9745788; doi:10.7717/peerj.14397)
Supplement: Figure S1 — A—Allele frequencies for all newly genotyped individuals from CH NW. B—Allele frequencies of the three re-genotyped individuals used by Kamm et al. (2009) from CH NE. Green—original genotypes, black—genotypes of re-genotyped individuals. C & D—Allele frequencies for F C and IT N, respectively. Green—original genotypes, gray—adjusted genotypes based on 12 re-genotyped individuals used by George et al. (2015). Numbers indicate the shift in base pairs (bp) for each marker. See Table 2 in the main text for details about the populations. [file peerj-10-14397-s005.docx]

**Figure S1** Allele frequency distribution and their respective adjustments. A – Allele frequencies for all newly genotyped individuals from CH NW. B – Allele frequencies of the three re-genotyped individuals used by *Kamm et al. (2009)* from CH NE. Green – original genotypes, black – genotypes of re-genotyped individuals. C & D – Allele frequencies for F C and IT N, respectively. Green – original genotypes, gray – adjusted genotypes based on 12 re-genotyped individuals used by *George et al. (2015)*. Numbers indicate the shift in base pairs (bp) for each marker. See Table 2 in the main text for details about the populations.
