# Supplementary material for: Cryptic population structure at the northern range margin of the service tree Sorbus domestica
Source: PeerJ. 2022 Dec 5;10:e14397. doi: 10.7717/peerj.14397 (PMC9745788; doi:10.7717/peerj.14397)
Supplement: Figure S2 — A—Optimum at K=2 for the dataset including all European samples (Fig. 1A). B—Optimum at K=2 for the dataset including samples from Switzerland and surrounding areas (Fig 1B). C—Optimum at K=2 for the dataset including Swiss samples only (Fig. 1C). [file peerj-10-14397-s006.docx]

**Figure S2** Delta K values on number of clusters K for the three datasets analyzed. A – Optimum at K=2 for the dataset including all European samples *(Fig 1A)*. B – Optimum at K=2 for the dataset including samples from Switzerland and surrounding areas (Fig 1B). C – Optimum at K=2 for the dataset including Swiss samples only *(Fig 1C)*.
